# Borneol for Regulating the Permeability of the Blood-Brain Barrier in Experimental Ischemic Stroke: Preclinical Evidence and Possible Mechanism

**DOI:** 10.1155/2019/2936737

**Published:** 2019-02-04

**Authors:** Zi-xian Chen, Qing-qing Xu, Chun-shuo Shan, Yi-hua Shi, Yong Wang, Raymond Chuen-Chung Chang, Guo-qing Zheng

**Affiliations:** ^1^Department of Neurology, The Second Affiliated Hospital and Yuying Children's Hospital of Wenzhou Medical University, Wenzhou, China; ^2^Laboratory of Neurodegenerative Diseases, School of Biomedical Sciences, LKS Faculty of Medicine, The University of Hong Kong, Hong Kong; ^3^State Key Laboratory of Brain and Cognitive Sciences, The University of Hong Kong, Hong Kong

## Abstract

Borneol, a natural product in the Asteraceae family, is widely used as an upper ushering drug for various brain diseases in many Chinese herbal formulae. The blood-brain barrier (BBB) plays an essential role in maintaining a stable homeostatic environment, while BBB destruction and the increasing BBB permeability are common pathological processes in many serious central nervous system (CNS) diseases, which is especially an essential pathological basis of cerebral ischemic injury. Here, we aimed to conduct a systematic review to assess preclinical evidence of borneol for experimental ischemic stroke as well as investigate in the possible neuroprotective mechanisms, which mainly focused on regulating the permeability of BBB. Seven databases were searched from their inception to July 2018. The studies of borneol for ischemic stroke in animal models were included. RevMan 5.3 was applied for data analysis. Fifteen studies investigated the effects of borneol in experimental ischemic stroke involving 308 animals were ultimately identified. The present study showed that the administration of borneol exerted a significant decrease of BBB permeability during cerebral ischemic injury according to brain Evans blue content and brain water content compared with controls (*P* < 0.01). In addition, borneol could improve neurological function scores (NFS) and cerebral infarction area. Thus, borneol may be a promising neuroprotective agent for cerebral ischemic injury, largely through alleviating the BBB disruption, reducing oxidative reactions, inhibiting the occurrence of inflammation, inhibiting apoptosis, and improving the activity of lactate dehydrogenase (LDH) as well as P-glycoprotein (P-GP) and NO signaling pathway.

## 1. Introduction

The blood-brain barrier (BBB) is an anatomical and biochemical barrier, consisting of endothelial cells, a basal lamina, and astrocytic end feet [1]. The BBB integrity is of great significance for brain homeostasis, while the dysfunction of BBB can lead to complications of neurological diseases, such as stroke, chronic neurodegenerative disorders, neuroinflammatory disorders, and brain tumor [1–3]. Moreover, a disruption of BBB integrity, characterized by increased permeability, is associated with several neurological pathologies, such as ischemia/hypoxia, hemorrhage, multiple sclerosis, and amyotrophic lateral sclerosis [4]. During the cerebral ischemia/reperfusion injury, changes in BBB structures result in the increase of its permeability and the loss of its protective function, which may deteriorate the tissue injury [5, 6]. Thus, the disruption of BBB is an essential pathological basis of cerebral ischemic injury. The long-lasting BBB disruption can directly contribute to cerebral edema and the influx of immune cell and inflammatory materials, ultimately resulting in the neuronal death, damage to the brain tissue, and neurological deficits, which plays a dominant role in the pathophysiological process of cerebral ischemic injury [7–10]. Further, extravasation of blood-borne albumin due to the increase of BBB permeability is assumed to trigger stroke-related complications like astrocyte-mediated epileptogenesis [11]. Consequently, those findings could provide a key basis that restore BBB integrity or reduce BBB opening is of great importance for the treatments of cerebral ischemic injury.

**Figure 1:**
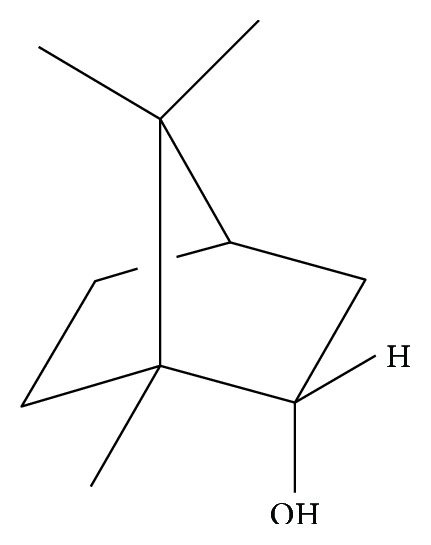
Chemical structures of borneol.

Borneol (Figure 1), a highly lipid-soluble bicyclic terpene chemical extracted from *Blumea balsamifera* (L.) DC. in the Asteraceae family or *Cinnamomum camphora* (L.) Presl. or chemically transformed based on camphor and turpentine oil [12], is early described in a Chinese herbology volume *The Compendium of Materia Medica* (Bencao Gangmu) during the 16^th^ century. It is widely used as a common ingredient in many traditional Chinese herbal formulas against stroke, such as Angong Niuhuang pill [13]. According to traditional Chinese medicine (TCM) Emperor-Minister-Assistant-Courier theory, borneol is classified as a “courier herb” that guides the herbs upward to target organ, especially in the upper part of the body, such as the brain. Many studies reported that borneol had neuroprotective effects through a variety of mechanisms like antinociception [14], anti-inflammatory [15, 16], antioxidation [16], and antiepilepsy [17]. Here, we conducted a preclinical systematic review to provide the current evidence of borneol for experimental ischemic stroke, mainly through possible mechanisms of regulating the BBB permeability.

## 2. Methods

### 2.1. Search Strategy

A comprehensive search strategy was conducted in seven databases, including PubMed, Embase, CENTRAL (the Cochrane Library), China National Knowledge Infrastructure, VIP database, Wanfang database, and Chinese Biomedical Database from their inceptions to July 2018. The search terms were as follows: “(borneol OR camphol) AND (ischemic stroke OR cerebral ischemic injury OR cerebral infarction OR brain infraction).” No restrictions were placed on the date, country, or language of publication. All searches were limited to animal studies.

### 2.2. Eligibility Criteria

#### 2.2.1. Types of Studies

All *in vivo* studies evaluating the effect of borneol on ischemic stroke were selected, regardless of animal species or publication status. The following eligibility criteria should be met: (1) the administration of borneol was performed on the animal model of ischemic stroke, regardless of its mode, dosage, and frequency; (2) the BBB integrity was assessed by qualitative assessments and/or quantitative evaluations of the brain or both the brain and the blood for a substance remaining stable in the blood or for an agent additionally injected into the blood, including brain water content, drugs used for ischemic stroke, Evans blue (EB), and imaging contrast agents; and (3) the control group receiving vehicle or no adjunct intervention was included in the studies.

#### 2.2.2. Types of Outcome Measures

The primary outcome measures were the brain EB content, the brain water content, and the ultrastructure of BBB. The secondary outcome measures were neurological function score (NFS), triphenyltetrazolium chloride (TTC) staining, measurement of superoxide dismutase (SOD) activity, and malonaldialdehyde (MDA) level.

### 2.3. Exclusion Criteria

Exclusion criteria were as follows: (1) the study was a case report, clinical trial, review, abstract, comment, editorial, or *in vitro* study; (2) the targeting disease was not ischemic stroke; (3) the intervention was a combination of borneol and another agent with potential effect on ischemic stroke; (4) the effect of borneol was not tested on BBB permeability; and (5) lack of the control group.

### 2.4. Data Extraction

Two independent reviewers assessed the articles for the eligibility and extracted the following details: (1) author, year, method of anesthesia, and/or model; (2) individual data, including animal species, sex, and weight; (3) the method of administration from both treatment and control groups, including drug, dose, mode, and frequency; and (4) the outcome measures and samples for individual comparison were included. A comparison was defined as the qualitative and/or quantitative assessments of the BBB permeability in the treatment and corresponding control groups after the administration of borneol or vehicle with a given dosage, mode, and frequency. If a drug was used for outcome assessment, both the drug and the method of drug administration were obtained. All available data from quantitative assessments of the BBB integrity were extracted for every comparison including mean outcome and standard deviation.

### 2.5. Quality of Evidence

Quality of evidence in included studies was conducted by two independent reviewers according to a ten-item modified scale [18, 19]: (1) peer reviewed publication; (2) statement of physiological parameter control, such as temperature; (3) random allocation; (4) blinded conduct of the experiments; (5) blinded assessment of outcome; (6) use of anesthetic without significant intrinsic neuroprotective activity; (7) appropriate animal and/or model (aged, diabetic, or hypertensive); (8) sample size calculation; (9) compliance with animal welfare regulations; (10) statement of potential conflict of interests.

### 2.6. Statistical Analysis

The statistical analysis was conducted via RevMan version 5.3. To estimate the effect of borneol on ischemic stroke, a model of random effect (RE) was applied to estimate pooled effect with 95% confidence intervals (CI) if statistical heterogeneity was found (*P* < 0.1, *I*^2^ > 50%), while a model of fixed effect (FE) was set with 95% CI if no statistical evidence of heterogeneity existed (*p* ≥ 0.1, *I*^2^ ≤ 50%). The weighted mean difference (WMD) was calculated as a summary statistic if the outcomes were applied the same scale, while standardized mean differences (SMD) was used if the same outcome measurements were measured in a variety of ways. Heterogeneity was assessed via a standard chi-square test and *I*^2^ statistic. A probability value less than 0.05 was considered statistically significant.

## 3. Results

### 3.1. Study Selection

**Figure 2:**
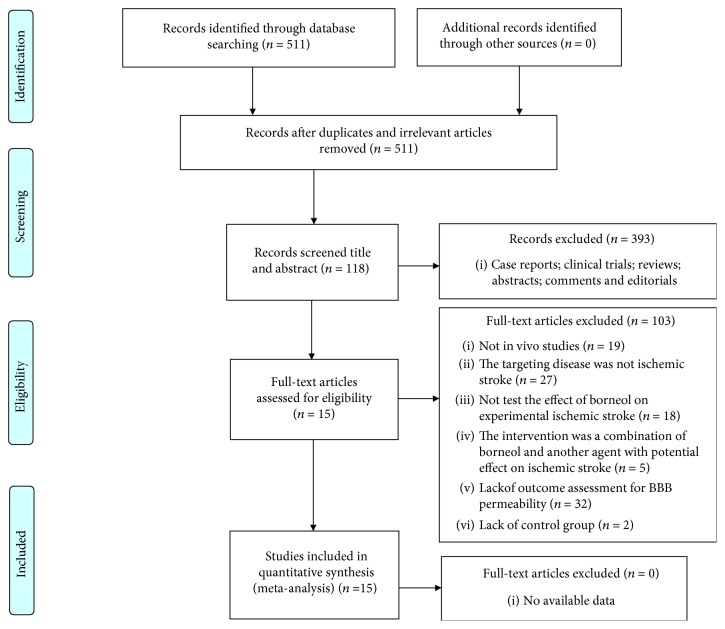
Flow diagram of the search process.

After primary search from seven databases, a total of 511 potentially relevant studies were included. By reviewing titles and abstracts, 393 studies that were case reports, clinical trials, reviews, abstracts, comments, or editorials were excluded. After reading the remaining 118 full-text articles for eligibility, 103 studies were removed for at least one of following reasons: (1) not an *in vivo* study; (2) the targeting disease was not ischemic stroke; (3) the study did not access the effects of borneol on the animal model of ischemic stroke; (4) the intervention was a combination of borneol and another agent with potential effect on ischemic stroke; (5) lack of outcome assessments for BBB integrity; and (6) lack of the control group. Ultimately, 15 studies involving 308 animals were selected for quantitative analysis (Figure 2).

### 3.2. Study Characteristics

Fifteen studies [20–34] involving 308 animals were included. Five species were referred, including SD rats (*n* = 225) [21–23, 25, 27–30, 33, 34], Wistar rats (*n* = 24) [24], ICR mice (*n* = 21) [33], Kunming mice (*n* = 14) [31], and C57 BL/6J mice (*n* = 24) [20]. The weight of rats ranged from 180 g to 400 g, and the weight of mice ranged from 18 g to 25 g. Cerebral ischemic injury in the included studies was induced by temporary middle cerebral artery occlusion (MCAO) for two hours [25–27, 30], permanent MCAO [21, 32], temporary occlusion of bilateral common carotid arteries for a range of 20-60 min [20, 22, 23, 33], transient occlusion of bilateral hemisphere for 20 min by Pulsinelli-4VO method [24], or permanent ligation of bilateral common carotid arteries [28, 29, 31, 34]. Chloral hydrate was used in twelve studies, while the anesthetic in three study [21, 26, 28] cannot be confirmed from the primary data.

Among all the included studies, one study [21] assessed the effects of L-borneol, D-borneol, and synthetic borneol, one study [23] assessed the effects of synthetic borneol as well as L-borneol, three studies [23, 29, 31] used synthetic borneol, one study [24] used D-borneol, two studies declared the administration of natural borneol [30] but without reporting the type of borneol, and the remaining studies used the borneol without further information provided. The mode of borneol application consisted of oral gavage (14 studies) and intravenous injection (1 study) [34]. The frequency of the treatment varied from once daily for the duration of 3 days [20, 21, 24, 26, 27, 29–31] to 8 days [33]. Two studies applied the borneol to the animals before as well as after the model establishment [20, 25], while the other thirteen studies applied it before the model establishment.

Four studies [23, 26–28] reported the ultrastructure of BBB when six studies performed the quantitative assessments of brain for EB [20, 24, 25, 30, 31], and ten studies performed the quantitative assessments of brain for water content [20, 22–25, 27, 29, 32–34]. More details about the characteristics of the included studies were shown in Table 1.

### 3.3. Quality of Included Study

The quality scores varied from 2/10 to 5/10 with the average of 3.13. Twelve studies were peer-reviewed publications, while three studies were unpublished master's thesis or PhD thesis. Four studies described the control of temperature. Fourteen studies declared the random allocation. Twelve studies described the use of anesthetic without significant intrinsic neuroprotective activity. Four studies stated the compliance with animal welfare regulations. One study had a statement of potential conflict of interests. None of the included studies reported the masked conduct of experiments, the blinded assessments of outcome, and the application of animal or model with relevant comorbidities or a sample size calculation. The quality scores for the included studies shown in Table 2.

### 3.4. Effectiveness Assessment

#### 3.4.1. The Brain EB Content

**Figure 3:**
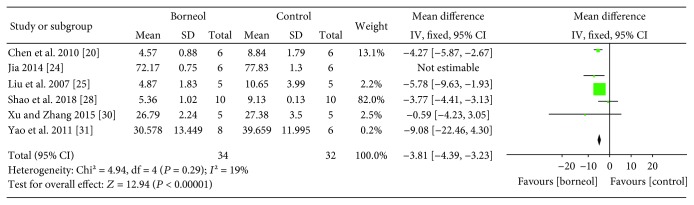
The forest plots: the borneol group versus the control group on brain Evans blue content under pathological conditions.

The assessments of the brain EB content were performed in six studies [20, 24, 25, 28, 30, 31] at a time point ranged from 20 min to 72 hours after the induction of the model. Combining available data in a meta-analysis from the above six studies showed the significantly protective effect of borneol on the BBB during cerebral ischemic injury according to the brain EB content [*n*_Treatment_/*n*_Control_ (*n*_T_/*n*_C_) = 40/38, WMD -4.16, 95% CI: -4.68~-3.64, *P* < 0.00001; heterogeneity *χ*^2^ = 12.34, df = 5, *I*^2^ = 59%]. After sequentially omitting each study, one outlier study [24] reporting Pulsinelli four-vessel method was considered as the potential sources of the heterogeneity. Meta-analysis of remaining four studies showed a more homogeneous result (*n*_T_/*n*_C_ = 34/32, WMD -3.81, 95% CI: -4.39~-3.23, *P* < 0.00001; heterogeneity *χ*^2^ = 4.94, df = 4, *I*^2^ = 19%, Figure 3). It was indicated that the method of model induction may be one possible explanation for the heterogeneity.

#### 3.4.2. The Brain Water Content

**Figure 4:**
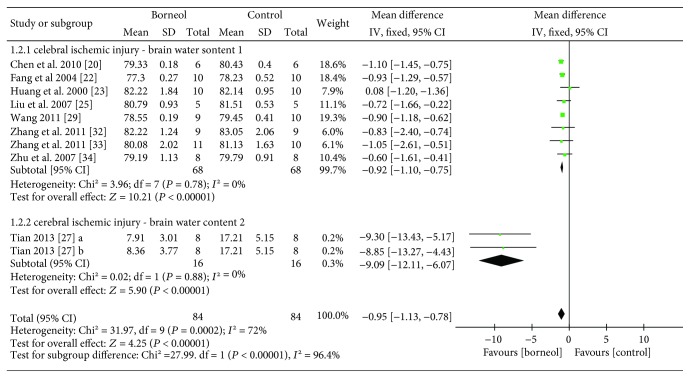
The forest plots: the borneol group versus the control group on brain water content under pathological conditions.

Ten studies [20, 22–25, 27, 29, 32–34] with eleven comparisons evaluated the brain water content at a time point ranged from 10 min to 72 hours after the induction of the model. Among them, nine studies assessed brain water content by using dry-wet weight method and showed the significant decreasing of BBB permeability in the treatment of cerebral ischemic injury (*n*_T_/*n*_C_ = 72/72, WMD -1.28, 95% CI: -1.93~-0.63, *P* = 0.0001; heterogeneity *χ*^2^ = 61.26, df = 8, *I*^2^ = 87%). After sequential removal of each study, the outlier study [24] with model induced by Pulsinelli four-vessel method was removed. The meta-analysis of eight studies showed a homogeneous result (*n*_T_/*n*_C_ = 68/68, WMD -0.92, 95% CI: -1.10~-0.75, *P* < 0.00001; heterogeneity *χ*^2^ = 3.96, df = 7, *I*^2^ = 0%, Figure 4), which also indicated a possible explanation for the heterogeneity. The remaining study [27] with two comparisons reported the rate of cerebral edema based on wet weight and meta-analysis of them showed significant effects of borneol for alleviating BBB permeability during cerebral ischemic injury (*n*_T_/*n*_C_ = 16/16, WMD -9.09, 95% CI: -12.11~-6.07, *P* < 0.00001; heterogeneity *χ*^2^ = 0.02, df = 1, *I*^2^ = 0%, Figure 4).

#### 3.4.3. The Ultrastructure of BBB

For cerebral ischemic injury, four studies [21, 23, 26, 27] with seven comparisons assessed the impacts of borneol on the ultrastructure of BBB, involving six comparisons reporting significantly neuroprotective effects and one comparison reporting no difference.

#### 3.4.4. NFS

NFS was reported in two studies [21, 32] with four comparisons, examined according to the five-point scale described previously by Longa et al. (Longa et al., 1989). Meta-analysis showed a significant difference in improving NFS but with substantial heterogeneity (*n*_T_/*n*_C_ = 45/45, MD -0.42, 95% CI: -0.65 to -0.20, *P* < 0.00001, heterogeneity *χ*^2^ = 300.05, df = 3, *I*^2^ = 99%). Zhang *et al*. [32] compared administration of borneol (0.4 g per animal, ig, qd) with the same volume of 1% Tween (ig, qd) for 7 days before occlusion and reported no significant difference in NFS between the two groups (*n*_T_/*n*_C_ = 9/9). Comparing L-borneol (0.2 g/kg, ig, qd), D-borneol (0.2 g/kg, ig, qd), or synthetic borneol (0.6 g/kg, ig, qd) with the same volume of 5% Tween 80 solution (ig, qd) for 3 days before occlusion, respectively, Dong et al. [21] found that these three comparisons had a significant difference in improving NFS (L-borneol: *n*_T_/*n*_C_ = 12/12; D-borneol: *n*_T_/*n*_C_ = 12/12; and synthetic borneol: *n*_T_/*n*_C_ = 12/12).

#### 3.4.5. TTC Staining

Two studies [21, 32] with four comparisons applied TTC staining to evaluate cerebral infarction area, showing a significant difference but with substantial heterogeneity (*n*_T_/*n*_C_ = 24/24, MD -6.64, 95% CI: -12.53 to -0.75, *P* < 0.00001, heterogeneity *χ*^2^ = 252.43, df = 3, *I*^2^ = 99%). Using borneol (0.4 g per animal, ig, qd) for 7 days before occlusion as an experimental administration (*n*_T_/*n*_C_ = 9/9), Zhang et al. [32] found that the infarction area was statistically similar between the borneol group and the control group (same volume of 1% Tween, ig, qd). Comparing L-borneol (0.2 g/kg, ig, qd), D-borneol (0.2 g/kg, ig, qd), or synthetic borneol (0.6 g/kg, ig, qd) with the same volume of 5% Tween 80 solution (ig, qd) for 3 days before occlusion, respectively, Dong *et al*. [21] found that these three comparisons had a significant difference in alleviating the infarction area (L-borneol: *n*_T_/*n*_C_ = 5/5; D-borneol: *n*_T_/*n*_C_ = 5/5; and synthetic borneol: *n*_T_/*n*_C_ = 5/5).

#### 3.4.6. SOD

**Figure 5:**
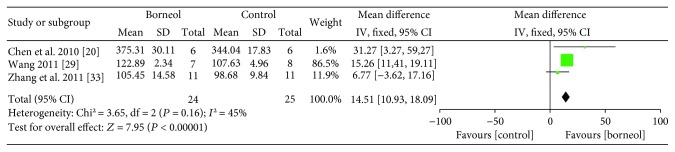
The forest plots: the borneol group versus the control group on SOD activity under pathological conditions.

Meta-analysis of four studies [20, 28, 29, 33] showed that animals in the borneol group had statistically significant higher SOD activity than the control group (*n*_T_/*n*_C_ = 34/35, MD 17.22, 95% CI: -10.00 to 24.44, *P* < 0.00001, heterogeneity *χ*^2^ = 20.56, df = 3, *I*^2^ = 85%). Sensitivity analyses were conducted to explore potential sources of heterogeneity after the omissions of each individual study from the original analysis. Sensitivity analyses pointed to one study [28] as a likely source of heterogeneity. After removal of the study, the SOD activity between the two groups had significant difference and the heterogeneity was reduced (*n*_T_/*n*_C_ = 24/25, MD 14.51, 95% CI: 10.93 to 18.09, *P* < 0.00001, heterogeneity *χ*^2^ = 3.65, df = 2, *I*^2^ = 45%, Figure 5).

#### 3.4.7. MDA

**Figure 6:**
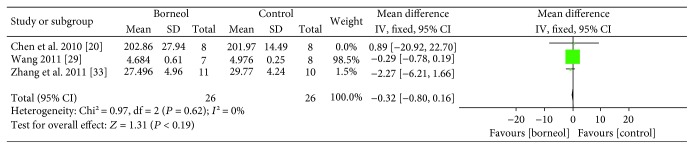
The forest plots: the borneol group versus the control group on MDA level under pathological conditions.

Three studies [20, 29, 33] based on the measurement of MDA level showed no significant difference between the borneol group and the control group but with substantial heterogeneity (*n*_T_/*n*_C_ = 24/24, WMD -0.39, CI: -6.25~1.63, *P* = 0.86, *I*^2^ = 66%). Sensitivity analyses were conducted to explore potential sources of heterogeneity after the omissions of each individual study from the original analysis. Sensitivity analyses pointed to one study [20] as a likely source of heterogeneity. After removal of the study, the MDA outcomes between the two groups remained similar and the heterogeneity was reduced (*n*_T_/*n*_C_ = 18/18, WMD -0.32, CI: -0.80~0.16, *P* = 0.19, *I*^2^ = 0%, Figure 6).

#### 3.4.8. Possible Neuroprotective Mechanisms of Borneol

According to the included studies, the possible neuroprotective mechanisms of borneol for ischemic stroke lie in the following aspects: (1) borneol could help alleviate the pathological BBB disruption [21, 23, 24, 26, 27, 30]. (2) Borneol could effectively reduce oxidative reactions through increasing the activity of SOD and decreasing the concentration of MDA [28, 29, 33]. (3) Borneol could inhibit the occurrence of inflammation by decreasing the expression of proinflammatory cytokines such as TNF-*α* [21, 28]. (4) Borneol could exert antiapoptotic effects, resulting in neuroprotection [21, 22]. (5) Borneol could improve the activity of lactate dehydrogenase (LDH) in brain tissue to inhibit the increase of lactic acid, which reduces the accumulation of lactic acid and exerts neuroprotective effect [29]. (6) Borneol could improve the energy metabolism disorder by upregulating the activity of Na^+^-K^+^-ATPase [27, 29], Ca^2+^-Mg^2+^-ATPase [27], and T-ATPase [27]. (7) Borneol could exert the neuroprotective effect via P-glycoprotein (P-GP) signaling pathway [27]. (8) Borneol exerts the neuroprotective effect via NO signaling pathway [27]. More details were shown in Table 3.

## 4. Discussion

### 4.1. Summary of Results

As far as we know, this is the first preclinical systematic review to determine the effects of borneol for experimental ischemic stroke, mainly through possible mechanisms of regulating the BBB permeability. In the present study, thirteen studies with 230 animals were selected for analysis. Borneol were identified to have a decreased impact on pathological BBB permeability from animals with cerebral ischemic injury. The pooled data suggested that borneol exerts a significant protection on the experimental model of ischemic stroke.

### 4.2. Limitations

First, all the databases we searched were in English or Chinese, which may cause selective bias as studies published in other languages may be left out. Second, the present study found that only one animal species (rodent) was used, which potentially posed a threat to the promotion of the findings. Third, the methodological quality of most included studies was moderate, which was an inherent drawback in the primary study. Methodological flaws in most included studies lie in blinding, sample size calculation, lacking animals with relevant comorbidities, and lacking statement of potential conflict of interests. Thus, the conclusions in the present study should be partially treated with caution.

### 4.3. Implications

Currently, it is increasingly recognized that the regulation of BBB permeability is a complicated process, which involves multiple components, such as endothelial cells, tight junction, basal lamina, and pericytes [35–38], and a variety of genes. BBB can effectively prevent the entry of lipophilic potential neurotoxins, protect the brain from most pathogens, and selectively transport essential molecules, which is of great importance in the homeostatic regulation of the brain microenvironment. As common pathological processes in many serious CNS diseases, the BBB destruction and the increasing BBB permeability are especially essential pathological bases of ischemic stroke. The present study demonstrated that the borneol could alleviate the increased BBB permeability by protecting the functions of endothelial cells and maintaining the integrity of the tight junction and basal lamina during cerebral ischemic injury, which exerts potential neuroprotective effect.

The possible neuroprotective mechanisms of borneol for ischemic stroke are summarized as follows: (1) BBB: borneol can alleviate the pathological BBB disruption through protecting the function of endothelial cells, maintaining the integrity of the basal lamina, and reducing the damage of tight junction integrity [23, 24, 26, 27, 30]. (2) Oxidative reactions: borneol can reduce oxidative reactions and the neurotoxicity of free radicals through increasing the activity of SOD and decreasing the concentration of MDA [29, 33]. SOD is a potent natural antioxidant enzyme that plays a bioprotective role by alternately catalyzing the dismutation or partitioning of the superoxide (O_2_-) radical into either ordinary molecular oxygen (O_2_) or hydrogen peroxide (H_2_O_2_), which is a prominent antioxidant defense in nearly all living cells exposed to oxygen [39]. As part of the brain ischemic antioxidant defense, an increase of SOD activities can ameliorate the oxidative stress damages [40]. MDA is a main product of lipid peroxidation of polyunsaturated fatty acids [41]. MDA can be overproduced, resulting from an increase in free radicals [42]. Thus, MDA is commonly used as a biomarker to measure the level of oxidative stress [43, 44]. Oxidative stress, including lipid peroxidation, plays an important role in the pathogenesis of acute brain injury and the breakdown of BBB [45]. Lipid peroxidation, mediated by superoxide, could cause alterations of membrane permeability as well as structural and functional impairment of cellular components. Free radicals are generally generated in the ischemic areas during ischemic stroke, leading to neuronal damage by promoting lipid peroxidation, protein breakdown, and DNA damage, which in turn results in cellular apoptosis and BBB permeability [46]. The pooled data showed that borneol significantly increases the activity of SOD and decreases the concentration of MDA which reduced the neurotoxicity of free radicals. (3) Anti-inflammation: borneol can exert anti-inflammation effects by decreasing the expression of proinflammatory cytokine TNF-*α* [21, 28]. Inflammation has been well recognized as a predominate contributor in ischemic stroke, playing an important role in all stages of the ischemic cascade [47, 48]. After ischemia, proinflammatory cytokines, such as TNF-*α*, were released, which resulted in BBB breakdown and neuronal death [21]. TNF-*α*, as a key proinflammatory mediator, plays a critical role in inflammatory responses. The present study indicated that borneol may be a potential anti-inflammatory drug, particularly in respect to cytokine suppression, resulting from the decreased expression of TNF-*α* induced by ischemic stroke. (4) Antiapoptosis: apoptosis is essential in the pathogenesis of acute and chronic neurodegenerative diseases, such as ischemic stroke, which can cause neuronal death and irreversible cerebral dysfunction [49]. It was found that inhibition of apoptosis could alleviate ischemic injury [50, 51]. The expression of Bcl-2 family plays a predominate role in apoptosis, which could either promote (Bax, Bak, Bad, Bim, and Bid) or prevent (Bcl-2, Bcl-XL, and Bcl-w) apoptosis [52, 53]. The present study revealed that borneol had antiapoptosis effects by decreasing the mRNA expression of Bax [22], increasing the mRNA expression of Bcl-XL [22] and modulating the Bax/Bcl-2 expression at both the mRNA and protein levels [21]. (5) LDH: LDH is an enzyme that is widely distributed in the cytoplasm of neurons and glial cells. LDH is abundant in the brain under physiological condition, which would release into the blood and consequently increase the accumulation of lactic acid during cerebral ischemic injury. The accumulation of lactic acid can cause acidosis in ischemic injury, which increases free radicals and decreases the use of glucose in the brain, ultimately resulting in the oxidative stress and the decrease in ATP levels [29]. Borneol can improve the activity of LDH to inhibit an increase in lactic acid [29]. (6) Energy metabolism: ischemic stroke easily causes the energy metabolism disorder including mitochondria functional impairment and imbalance of ion homeostasis [54]. Borneol can improve the energy metabolism disorder through upregulating the activity of Na^+^-K^+^-ATPase [27, 29], Ca^2+^-Mg^2+^-ATPase [27], and T-ATPase [27]. (7) P-glycoprotein (P-GP): P-GP is an important protein of the cell membrane and more accurately an ATP-dependent efflux pump with broad substrate specificity. P-GP expressed in the capillary endothelial cells composes the BBB [55–57]. Borneol could decrease the expression of P-GP. (8) NO: excessive nitric oxide (NO) generated during the cerebral ischemic stroke may be combined with free radicals, which causes damage to lipid membranes, nucleic acids, and cell protein. Borneol exerts the neuroprotective effect via NO signaling pathway [27]. Borneol could decrease the expression of NO [27]. During cerebral ischemia and reperfusion, signaling cascades can be triggered by cross talk. As mentioned above, BBB disruption, oxidative stress, acidosis, the energy metabolism disorder, P-GP, and NO can affect each other and interact as both cause and effect. The present study showed the neuroprotective mechanism of borneol in the treatment of cerebral ischemic injury was mainly attributed to the decrease of BBB permeability. However, cellular and molecular alteration mechanisms of borneol for ischemic stroke have not been clearly elucidated yet, which presented an exciting investigative direction of further research in this field. In addition, the administration of borneol could expand to other diseases with similar pathologies, namely, the increased BBB permeability, which was associated with the development and progression of many CNS diseases, such as multiple sclerosis, Alzheimer's disease, and HIV-associated dementia.

Infarct volume (IV) is a common index for assessing the extent of cerebral ischemic injury. Infarct on imaging is one of the common identified predictors of clinical outcome for ischemic stroke [58]. In addition, IV measurement is clinically helpful in the accurate selection of patients for decompressive surgery and the determination of the time of surgery [59]. Therefore, it is essential for further experimental trails of borneol for ischemic stroke to choose IV as outcome measures.

The methodological quality of the included studies was moderate. In particular, no study estimated the sample size, since inadequate sample size can miss the real intervention effect in an experiment or excessive sample size can result in wasting animals and raising animal ethical issues [60]. No study blindingly assessed outcome, which could attribute to a 27% overestimation of the mean reported effect size [61]. No study used animals with relevant comorbidities, which was rarely like human pathology under the clinical conditions [18]. Thus, it is necessary for further research of borneol for ischemic stroke to take a rigor experimental design into consideration. We recommended that the Animal Research: Reporting of In Vivo Experiments (ARRIVE) [62], a reporting guideline consisting of a 20-item checklist for the introduction, methods, results, and discussion, should be used as guidelines for the design and reporting of further experimental research examining borneol for ischemic stroke, which can extremely help improve the methodological quality.

## 5. Conclusion

Borneol can alleviate the BBB disruption and plays a protective role on cerebral ischemic injury through multiple signaling pathways. Further relevant molecular mechanisms deserve adequate research.

## Figures and Tables

**Table 1:** Summary of the efficacy of borneol for ischemic stroke.

Study (author, years)	Species	Weight	Anesthetic	Conditions or model induction	Method of administration		Outcome measures (sample)	Intergroup differences
					Treatment group	Control group		
Chen et al. 2010 [20]	Male, C57 BL/6J mice	18–20 g	Chloral hydrate (300 mg/kg, ip)	Temporary occlusion of bilateral common carotid arteries for 25 min	Borneol, 0.01 g/kg, ig, at 30 min before model and once daily for 2 days after occlusion	Same volume of 10% ethanol, ig, at 30 min before model and once daily for 2 days after occlusion	(1) Brain EB content, 48 h (6/6) (2) Brain water content, 24 h (6/6) (3) Morris water maze test, 4 d (12/12) (4) MDA, SOD, 4 d (6/6) (5) GFAP, 4 d (3/3)	(1) *P* < 0.05 (2) *P* < 0.05 (3) *P* > 0.05 (4) *P* > 0.05
Dong 2018 [21] a	Male, SD rats	230–270 g	NS	Permanent middle cerebral artery occlusion	L-borneol, 0.2 g/kg, ig, once daily for 3 days before occlusion	Same volume of 5% Tween 80 solution, ig, once daily for 3 days before occlusion	(1) Neurological function score, 24 h (12/12) (2) Brain water content, 24 h (8/8) (3) Cerebral infarction rate with TTC staining, 24 h (5/5) (4) The ultrastructure of BBB, NS (5) VEGF levels in the serum, 24 h (7/7) (6) TNF-*α* levels in the serum, 24 h (7/7) (7) Bax mRNA, 24 h (3/3) (8) Bcl-2 mRNA, 24 h (3/3) (9) Claudin-5 mRNA, 24 h (3/3)	(1) *P* < 0.05 (2) *P* < 0.01 (3) *P* < 0.01 (4) NS (5) *P* < 0.01 (6) *P* < 0.01 (7) *P* > 0.05 (8) *P* > 0.05 (9) *P* > 0.05
Dong 2018 [21] b	Male, SD rats	230–270 g	NS	Permanent middle cerebral artery occlusion	D-borneol, 0.2 g/kg, ig, once daily for 3 days before occlusion	Same volume of 5% Tween 80 solution, ig, once daily for 3 days before occlusion	(1) Neurological function score, 24 h (12/12) (2) Brain water content, 24 h (8/8) (3) Cerebral infarction rate with TTC staining, 24 h (5/5) (4) The ultrastructure of BBB, NS (5) VEGF levels in the serum, 24 h (7/7) (6) TNF-*α* levels in the serum, 24 h (7/7) (7) Bax mRNA, 24 h (3/3) (8) Bcl-2 mRNA, 24 h (3/3) (9) Claudin-5 mRNA, 24 h (3/3)	(1) *P* > 0.05 (2) *P* > 0.05 (3) *P* > 0.05 (4) NS (5) *P* < 0.01 (6) *P* < 0.01 (7) *P* > 0.05 (8) *P* > 0.05 (9) *P* < 0.05
Dong 2018 [21] c	Male, SD rats	230–270 g	NS	Permanent middle cerebral artery occlusion	Synthetic borneol, 0.6 g/kg, ig, once daily for 3 days before occlusion	Same volume of 5% Tween 80 solution, ig, once daily for 3 days before occlusion	(1) Neurological function score, 24 h (12/12) (2) Brain water content, 24 h (8/8) (3) Cerebral infarction rate with TTC staining, 24 h (5/5) (4) The ultrastructure of BBB, NS (5) VEGF levels in the serum, 24 h (7/7) (6) TNF-*α* levels in the serum, 24 h (7/7) (7) Bax mRNA, 24 h (3/3) (8) Bcl-2 mRNA, 24 h (3/3) (9) Claudin-5 mRNA, 24 h (3/3)	(1) *P* > 0.05 (2) *P* < 0.01 (3) *P* < 0.01 (4) *P* < 0.01 (5) *P* < 0.01 (6) *P* < 0.01 (7) *P* < 0.05 (8) *P* > 0.05 (9) *P* < 0.01
Fang et al. 2004 [22]	Male and female, SD rats	200–300 g	10% chloral hydrate (300 mg kg, ip)	Temporary occlusion of bilateral common carotid arteries for 60 min	Borneol, 0.2 g/kg, ig, once daily for 7 days before occlusion	Same volume of normal saline, ig, once daily for 7 days before occlusion	(1) Brain water content, NS (10/10) (2) BAX, NS (10/10) (3) BCL-XL, NS (10/10)	(1) *P* < 0.001 (2) *P* < 0.001 (3) *P* < 0.05
Huang et al. 2000 [23]	Male and female, SD rats	180–200 g	NS	Temporary obstruction of bilateral common carotid arteries for 45 min	Synthetic borneol, 1 g/kg, ig, once daily for 7 days before model establishment	Same volume of distilled water, ig, once daily for 7 days before model establishment	(1) Brain water content, 1 h (10/10) (2) The ultrastructure of BBB, 1 h (10/10)	(1) *P* > 0.05 (2) NS
Jia 2014 [24]	Male, Wistar rats	240–280 g	10% chloral hydrate (NS, ip)	Transient occlusion of bilateral hemisphere for 20 min (Pulsinelli-4VO method)	D-borneol, 0.2 g/kg, ig, once daily for 3 days before occlusion	Same volume of 70% ethanol, ig, once daily for 3 days before occlusion	(1) Brain EB content, 72 h (6/6) (2) Brain water content, 72 h (6/6) (3) ZO-1, 72 h (6/6)	(1) *P* < 0.05 (2) *P* < 0.05 (3) *P* < 0.05
Liu et al. 2007 [25]	Male and female, SD rats	260–300 g	10% chloral hydrate (350 mg/kg, ip)	Temporary middle cerebral artery occlusion for 2 h	Borneol, 0.003 g/kg, ig, at 12 h, 30 min before model and at 12 h, 24 h after occlusion	Same volume of 1% Tween, ig, at 12 hr, 30 min before model and at 12 hr, 24 hr after occlusion	(1) Brain EB content, 24 h (5/5) (2) Brain water content, 24 h (5/5)	(1) *P* < 0.01 (2) *P* > 0.05
Ni et al. 2011 [26]	Male, SD rats	280–320 g	10% chloral hydrate (350 mg/kg, ip)	Temporary middle cerebral artery occlusion for 2 h	Borneol, 0.2 g/kg, ig, once daily for 3 days before occlusion	Same volume of 5% Tween, ig, once daily for 3 days before occlusion	(1) The ultrastructure of BBB, 22 h (NS/NS) (2) VEGF, 22 h (8/8) (3) MMP-9, 22 h (8/8)	(1) NS (2) *P* < 0.05 (3) *P* > 0.05
Tian 2013 [27] a	Male, SD rats	280–350 g	10% chloral hydrate (350 mg/kg, ip)	Temporary middle cerebral artery occlusion for 2 h	L-borneol, 0.20 g/kg, ig, once daily for 3 days before occlusion	Same volume of normal saline, ig, once daily for 3 days before occlusion	(1) Rate of cerebral edema, 22 h (8/8) (2) MDA, 22 h (8/8) (3) SOD, 22 h (8/8) (4) P-GP, 22 h (8/8) (5) The ultrastructure of BBB, 22 h (2/2)	(1) *P* < 0.01 (2) *P* < 0.05 (3) *P* < 0.01 (4) *P* < 0.01 (5) NS
Tian 2013 [27] b	Male, SD rats	280–350 g	10% chloral hydrate (350 mg/kg, ip)	Temporary middle cerebral artery occlusion for 2 h	Synthetic borneol, 0.20 g/kg, ig, once daily for 3 days before occlusion	Same volume of normal saline, ig, once daily for 3 days before occlusion	(1) Rate of cerebral edema, 22 h (8/8) (2) MDA, 22 h (8/8) (3) SOD, 22 h (8/8) (4) P-GP, 22 h (8/8) (5) The ultrastructure of BBB, 22 h (2/2)	(1) *P* < 0.01 (2) *P* < 0.05 (3) *P* < 0.01 (4) *P* < 0.01 (5) NS
Shao 2018 [28]	Male & female, SD rats	300–400 g	NS	Permanent ligation of bilateral common carotid arteries	Borneol, 0.5 g/kg, ig, once daily for 7 days before model establishment	Same volume of normal saline, ig, once daily for 7 days before model establishment	(1) Brain EB content, 24 h (10/10) (2) SOD, NS (10/10) (3) MPO, NS (10/10) (4) TNF-*α*, NS (10/10)	(1) *P* < 0.01 (2) *P* < 0.01 (3) *P* < 0.01 (4) NS
Wang 2011 [29]	Male and female, SD rats	180–220 g	10% chloral hydrate (300 mg/kg, ip)	Permanent ligation of bilateral common carotid arteries	Synthetic Borneol, 0.2 g/kg, ig, once daily for 3 days before model establishment	Same volume of normal saline, ig, once daily for 3 days before model establishment	(1) Brain water content, 3 h (9/10) (2) SOD, Na^+^-K^+^-ATPase, 3 h (9/10) (3) MDA, 3 h (9/10) (4) LDH, 3 h (9/10)	(1) *P* > 0.05 (2) *P* < 0.05 (3) *P* < 0.05 (4) *P* < 0.05
Xu and Zhang 2015 [30]	Male, SD rats	250 ± 20 g	10% chloral hydrate (NS, NS)	Temporary middle cerebral artery occlusion for 2 h	Natural borneol, 0.028 g/kg, ig, once daily for 3 days before occlusion	Same volume of normal saline, ig, once daily for 3 days before occlusion	(1) Brain EB content, 24 h (5/5) (2) ZO-1, 24 h (5/5) (3) Claudin-5, 24 h (5/5)	(1) *P* > 0.05 (2 *P* > 0.05 (3) *P* > 0.05
Yao et al. 2011 [31]	Male and female, Kunming mice	22 ± 3 g	4% chloral hydrate (400 mg/kg, ip)	Permanent ligation of bilateral common carotid arteries	Synthetic borneol, 0.0666 g/kg, ig, once daily for 3 days before occlusion	Same volume of normal saline, ig, once daily for 3 days before occlusion	Brain EB content, 20 min (8/6)	*P* > 0.05
Zhang et al. 2011 [32]	Male, SD rats	250–280 g	10% chloral hydrate (350 mg/kg, ip)	Permanent middle cerebral artery occlusion	Borneol, 0.4 g per animal, ig, once daily for 7 days before occlusion	Same volume of 1% Tween, ig, once daily for 7 days before occlusion	(1) Brain water content, 24 h (9/9) (2) Neurological function score, NS (9/9) (3) Infarction volume, NS (9/9)	(1) *P* > 0.05 (2) *P* > 0.05 (3) *P* > 0.05
Zhang et al. 2011 [33]	Male and female, ICR mice	18–22 g	4% chloral hydrate (0.4 mg/kg, NS)	Temporary occlusion of bilateral common carotid arteries for 20 min	Borneol, 0.5 g per animal, ig, once daily for 8 days before occlusion	Same volume of 1% Tween, ig, once daily for 8 days before occlusion	(1) Brain water content, 10 min (11/10) (2) SOD, 10 min (11/10) (3) MDA, 10 min (11/10)	(1) *P* > 0.05 (2) *P* > 0.05 (3) *P* > 0.05
Zhu et al. 2007 [34]	Male, SD rats	200–250 g	10% chloral hydrate (400 mg/kg, ip)	Permanent ligation of bilateral common carotid arteries	Borneol, 0.020 g/kg, iv, once daily for 4 days before model establishment	Same volume of normal saline, iv, once daily for 4 days before model establishment	Brain water content, 3 h (8/8)	*P* > 0.05

Note: BBB: the blood-brain barrier; increased: a significantly increasing blood-brain barrier permeability after the administration of borneol; decreased: a significantly decreasing blood-brain barrier permeability after the administration of borneol; ND: no statistical difference between treatment and control groups; Increased? or decreased?: the efficacy result was reported as increasing or decreasing blood brain barrier permeability with the absence of statistical analysis or available original data; ig: intragastric administration; ip: intraperitoneal administration; iv: intravenous injection; NS: not stated; SOD: superoxide dismutase; MDA: malondialdehyde; ET: endothelin; NO: nitric oxide; LDH: lactated hydrogenase; EB: Evans blue; ZO-1: zonula occludens-1; GFAP: gliofibrillar acid protein; BAX: Bcl-2 associated X protein; BCL: B-cell lymphoma; VEGF: vascular endothelial growth factor; MMP-9: matrix metalloproteinase-9; P-GP: P-glycoprotein.

**Table 2:** Quality assessment of included studies.

Study	A	B	C	D	E	F	G	H	I	J	Total
Chen et al. [20]	+	+	+			+			+		5
Dong et al. [21]	+	+	+						+	+	5
Fang et al. [22]	+		+			+					3
Huang et al.[23]	+		+								2
Jia [24]			+			+			+		3
Liu et al. [25]	+		+			+					3
Ni et al. [26]	+					+			+		3
Tian [27]		+	+			+					3
Shao et al. [28]	+		+								2
Wang [29]			+			+					2
Xu and Zhang [30]	+		+			+					3
Yao et al. [31]	+		+			+					3
Zhang et al. [32]	+	+	+			+					4
Zhang et al. [33]	+		+			+					3
Zhu et al. [34]	+		+			+					3

A: peer-reviewed publication; B: monitoring of physiological parameters such as temperature; C: random allocation; D: blinded conduct of the experiments; E: blinded assessment of outcome; F: use of anesthetic without significant intrinsic neuroprotective activity (e.g., ketamine); G: animal and/or model (aged, diabetic, or hypertensive); H: sample size calculation; I: compliance with animal welfare regulations; J: statement of potential conflict of interests.

**Table 3:** Characteristics of mechanism studies of borneol on experimental ischemic stroke.

Study	Model	Method of administration (experimental group versus control group)	Possible mechanism
Dong et al. [21]	pMCAO in SD rats	(1) L-borneol versus 5% Tween 80 (2) D-borneol versus 5% Tween 80 (3) Synthetic borneol versus 5% Tween 80	(1) Alleviate the pathological BBB disruption by upregulating tight junction proteins Claudin-5 (2) Accelerate the proliferation of vascular endothelial cells and by initiating angiogenesis. (3) Anti-inflammation by decreasing the expression of TNF-*α* (4) Antiapoptosis by modulating the Bax/Bcl-2 expression at both the mRNA and protein levels
Fang et al. [22]	BCO/1 h in SD rats	Borneol versus normal saline	Antiapoptosis by decreasing the mRNA expression of Bax and increasing the mRNA expression of Bcl-XL
Huang et al. [23]	BCO/45 min in SD rats	Synthetic borneol versus distilled water	Alleviate the pathological BBB disruption by protecting the function of endothelial cells and maintaining the integrity of the basal lamina
Jia [24]	Occlusion of bilateral hemisphere (Pulsinelli-4VO method)/20 min in Wistar rats	D-borneol versus 70% ethanol	Alleviate the pathological BBB disruption by upregulating tight junction proteins ZO-1
Ni et al. [26]	MCAO/2 h in SD rats	Borneol versus 5% Tween	Alleviate the pathological BBB disruption by downregulating VEGF and MMP-9
Tian [27]	MCAO/2 h in SD rats	(1) L-borneol versus normal saline (2) Synthetic borneol versus normal saline	(1) Alleviate the pathological BBB disruption by alleviating the damage of the BBB tight junction integrity (2) Reduce oxidative reactions by increasing the activity of SOD and decreasing the concentration of MDA (3) Improve the energy metabolism disorder by upregulating the activity of Na^+^-K^+^-ATPase, Ca^2+^-Mg^2+^-ATPase, and T-ATPase (4) Neuroprotection via P-GP signaling pathway (5) Neuroprotection via NO signaling pathway
Shao et al. [28]	Permanent BCO in SD rats	Borneol versus normal saline	(1) Reduce oxidative reactions by increasing the activity of SOD (2) Anti-inflammation by decreasing the expression of TNF-*α*
Wang [29]	Permanent BCO in SD rats	Synthetic borneol versus 1% Tween	(1) Reduce oxidative reactions by increasing the activity of SOD and decreasing the concentration of MDA (2) Improve the activity of LDH (3) Improve the energy metabolism disorder by upregulating the activity of Na^+^-K^+^-ATPase
Xu and Zhang [30]	MCAO/2 h in SD rats	Borneol versus normal saline	Alleviate the pathological BBB disruption by upregulating tight junction proteins ZO-1 and claudin-5
Zhang et al. [33]	BCO/20 min in ICR mice	Borneol versus 1% Tween	Reduce oxidative reactions by increasing the activity of SOD and decreasing the concentration of MDA

Note: BCO, bilateral carotid occlusion; MCAO, middle cerebral artery occlusion; pMCAO, permanent middle cerebral artery occlusion; LDH, lactate dehydrogenase; ZO-1, Zonula occludens-1; VEGF, vascular endothelial cell growth factor; MMP-9matrix metalloproteinase-9; P-GP, P-glycoprotein.
